# Real-Time Neural Signals of Disorder and Order Perception

**DOI:** 10.3389/fpsyg.2019.00357

**Published:** 2019-02-22

**Authors:** Kaiyun Li, Huijing Yang, Xiaoning Qi, Fengxun Lin, Gongxiang Chen, Minfang Zhao

**Affiliations:** ^1^School of Education and Psychology, University of Jinan, Jinan, China; ^2^School of Education Science, Huizhou University, Huizhou, China

**Keywords:** disorder, order, cognitive disfluency, physical environmental and basic visual pictures, ERPs

## Abstract

Order and disorder are prevalent in everyday life, yet little is known about the neural real-time processing that occurs during the perception of disorder relative to order. In the present study, from a cognitive perspective, by adopting the ERP method, we aimed to examine the elicited real-time neural signals of disorder and order perception when participants processed physical environmental and basic visual disorder and order pictures in an irrelevant red or green rectangle detection task, and we attempted to test the hypothesis of cognitive disfluency in disorder perception. Generally, we observed that at each measured time interval, the ERPs elicited by order stimuli were more positive (less negative) in amplitude than those elicited by disorder stimuli at the frontal electrodes (represented by F7/F8, FT7/FT8, Fz, and FCz), whereas at the posterior electrodes (represented by P7/P8, PO7/PO8, Pz, and POz), the opposite was true. These data reveal for the first time the neural underpinnings of disorder and order perception, extending our understanding of the nature of disorder and order. This study also contributes to the cognitive fluency literature and indirectly expands the research on disorder and order stimuli in cognitive fluency.

## Introduction

Order and disorder are prevalent in everyday life, both in the home and in the workplace (Koole and Van den Berg, 2005), as well as in culture (Baumeister, 2005). People are drawn to order instinctively since it is comforting because of its association with predictability, which could allow people to confidently pursue goals and effectively interact with their environment (Harmonjones and Harmonjones, 2002). Disorder, in contrast, can be revoltive because it prevents people from foresighted what will happen next (Hirsh et al., 2012; Kotabe, 2014; Tullett et al., 2015). Therefore, order and disorder situations might be functional, particularly insofar as they activate different psychological states and facilitate different types of outcomes.

Considerable evidence from different areas of scientific inquiry has suggested that in contrast to perceived order, perceived disorder apparently has a variety of detrimental psychological and behavioral consequences. For example, exposure to disorderly environments can elicit negative effects, including perceived powerlessness (Geis and Ross, 1998) and distress (Cutrona et al., 2000), feeling unsafe (Perkins et al., 2010), depression (Ross, 2000), and anxiety and performance monitoring (Tullett et al., 2015); can diminish a sense of meaning in life (Heintzelman et al., 2013) and reduce self-control and cognitive control (Chae and Zhu, 2014); and can also encourage delinquency, rule breaking and criminal behavior (Keizer et al., 2008; Kotabe et al., 2016b). Obviously, the previous research on disorder has tended to focus on its consequences, yet little is known about the neural real-time processing that occurs during the perception of disorder relative to order. To the best of our knowledge, until now, no work has shown the real-time neural signals related to disorder and order perception. Event-related potentials (ERPs) are measures of brain electrical activity (i.e., EEGs) that are recorded from multiple locations across the scalp, time locked to the presentation of a stimulus, and averaged to reveal the typical activity corresponding to the cognitive processes under investigation (Luck et al., 2000). Thus, by adopting the ERP method, the present research sought to explore real-time neural signals during the processing of images of disorder and order in an irrelevant red or green rectangle identification task.

The general problem in previous studies involving disorder perception is that “disorder” and “order” have not been clearly defined or assessed (Harcourt, 2009). One of the specific issues is that previous research has confounded social disorder and visual disorder. Social disorder typically refers to variation in the real environment, such as that due to the presence versus absence of litter. Accordingly, previous field experiments and laboratory experimental methods usually created situations of social disorder (in real and virtual environments) to examine whether and how disorderly environments encourage an individual’s impulsive and disorderly behaviors (Braga et al., 1999; Braga and Bond, 2010; Vohs et al., 2013; Chae and Zhu, 2014). For example, Heintzelman et al. (2013) manipulated perceived disorder either by (a) presenting people with pictures of seasons in temporal sequence (e.g., autumn, winter, spring, summer) (order manipulation), in random sequence (e.g., winter, autumn, summer, spring; Experiments 1 and 2) (disorder manipulation); or (b) presenting people with semantic triads (i.e., Remote Associates Test items) that were either coherent (e.g., “falling, actor, dust”; common associate: star) or incoherent (e.g., “belt, deal, nose”; Experiments 3 and 4). Researchers manipulated perceived disorder after the manner of Vohs et al. (2013) – by having people perform tasks in either a disorderly or orderly lab environment. Undoubtedly, these operationalizations of disorder/order were not satisfied with the technical parameters for measuring neural activity, such as those of the ERP and fMRI techniques.

**FIGURE 1:**
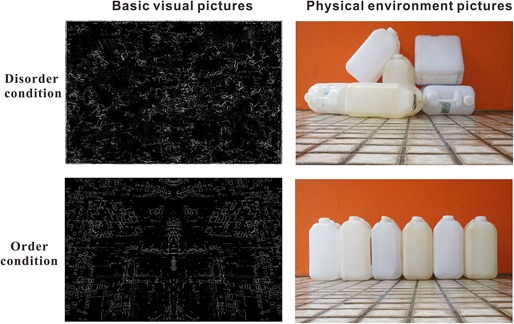
Illustration of the stimuli: basic visual disorder/order pictures and physical environmental disorder/order pictures.

Sampson and Raudenbush (2004) first attempted to separate a “physical disorder/order” component from environmental social disorder, and the operationalization of “physical disorder or order” was measured by three questions: “How much of a problem is/are litter/trash, graffiti, and vacant housing/storefronts [in your neighborhood]?)”. Recently, Kotabe (2014), from a cognitive perspective, operationalized the definition of perceived environmental social disorder and order as follows: “Perceived disorder is an interpreted state of the world in which things are in non-patterned and non-coherent positions. In contrast, perceived order is an interpreted state of the world in which things are in patterned and coherent positions”. Therefore, the key requirement of “physical disorder/order” is that the degree of the stimulus is processed based on “orderliness, regularity and pattern and the rationality of its place”. Based on Kotabe’s definition, Peng (2017) created a set of pictures of physical environmental disorder by depicting places or objects artificially arranged in non-patterns and non-coherent patterns; conversely, pictures of the physical environmental order depicted places or objects artificially arranged in patterns and coherent patterns. In the current study, we attempted to measure the time processing of physical environment disorder and order by adopting the pictures used in Hu’s study as the experimental stimuli (see the illustration in Figure 1).

In the real world, a scene of an environment usually contains low-level visual features (e.g., edges, colors, spatial frequency) and high-level semantic features (e.g., places and objects) (Rosch and Mervis, 1975; Oliva and Schyns, 2000; Oliva, 2005; Oliva and Torralba, 2006). The aforementioned “physical disorder/order” stimuli involved high-level or semantic information. Kotabe et al. (2016a,b) recently proposed a method to distinguish the basic visual cues from the high-level cues in physical environmental disorder/order. They defined “visual disorder” as the perception of disorder attributable to basic (or low-level) visual features (i.e., spatial and color features, basic visual disorder cues). Adopting a principled approach to reconstructed stimuli contrasted in terms of visual disorder but lacking scene-level or semantic (referring to meaningful information such as that involved in the recognition of objects, places, and general descriptors) disorder cues, they found that spatial features (e.g., non-straight edges, asymmetry) were most important for visual disorder/order (see the illustration in Figure 1). Kotabe et al. (2016a) further reported that even basic visual disorder cues (simple spatial perceptual properties of the environment) could affect complex behavior (e.g., cheating, rule-breaking), consistent with the results of social disorder (Keizer et al., 2008). Therefore, in the current study, using 60 basic visual disorder/order pictures as the experimental stimuli, which were reconstructed from and used in Kotabe et al. (2016a) study, we further attempted to measure the brain neural activity in basic visual disorder/order perception by adopting the ERP method.

A key process in early visual processing in the visual areas concerns low-level stimulus features, including luminance, spatial frequency, and orientation. An earlier negative or positive evoked potential at posterior occipital, temporal, and parietal electrodes at approximately 100 ms (e.g., from 60 to 100 ms) has been shown to reflect luminance (Johannes et al., 1995), spatial frequency (Kenemans et al., 2000), orientation (Arakawa et al., 2000), and size and eccentricity differences (Busch et al., 2004). In the current study, there was good reason to expect that both disorder and order pictures would elicit early waveforms that were either negative or positive at approximately 100 ms at the posterior electrodes. However, it should be noted that there might be no significant divergence at approximately 100 ms at the posterior electrodes between the order and disorder pictures conditions since the low-level features were well matched in the current study between the disorder and order pictures.

Perceived disorder might be cognitively processed more disfluently than perceived order (Kotabe, 2014). Disfluency is believed to cause people to think more deeply and abstractly (Alter, 2013). Kotabe et al. (2016a) further proposed that visually disordered stimuli were more redundant and conveyed more information than visually ordered stimuli. These aspects could render viewing visually disordered stimuli more cognitively demanding than viewing visually ordered stimuli at the high processing level (see, Kinchla, 1977; Field, 1987; Olshausen and Field, 1996). In the current study, cognitive processing disfluency in the disorder condition or processing fluency in the order condition might be caused or reflected mainly by perceptual disfluency/fluency, referring to the ease of processing stimuli that arises from variations in perceptual quality (e.g., clarity) (Reber et al., 2004; Leynes and Zish, 2012). There have been a few key studies suggesting that perceptual fluency ERP effects occur earlier (approximately 100 to 400 ms) at the frontal and posterior scalp locations (e.g., Nessler et al., 2005; Woollams et al., 2008; Voss and Paller, 2010; Kurilla and Gonsalves, 2012). However, the pattern of ERPs elicited by fluent and disfluent stimuli remains controversial. Most studies, for example, Nessler et al. (2005); Woollams et al. (2008), and Kurilla and Gonsalves (2012), have found that more fluent stimuli were associated with more positive ERPs, whereas Voss and Paller (2010) reported that more fluent stimuli elicited a more negative ERP. Two works of Leynes found that the pattern of ERPs elicited by the fluent and disfluent stimuli was influenced by the sequence of stimuli presentation. For example, in one work by Leynes and Zish (2012), the clarity of words was manipulated to create the experience of disfluency or fluency. The results revealed that, only at the posterior electrodes, ERPs differed between clear and blurry probes (words) when the clarity was blocked (one block of clear products and one block of blurry products) but not when the clarity varied randomly across trials, and the ERP effect observed during the blocked test indicated that the more fluent stimulus (clear words) elicited a more negative (less positive) ERP than the disfluent stimulus (blurred words). Leynes and Addante (2016) further directly compared the ERPs between clear and blurry photographs of off-brand products and found that at the frontal and parietal electrodes, when the presentation of the blurred and clear images varied trial by trial according to a random trial sequence, the blurred images (disfluent) elicited more positive (less negative) ERPs than the clear images (fluent) in the time window of 100–800 ms, whereas when the presentation of the blurred and clear images varied across trial blocks, the blurred images elicited more negative ERPs than the clear images in the time window of 100–800 ms. In the current study, for both the basic visual stimuli (presented in the first block) and the physical environmental stimuli (presented in the second block), the presentation of the disorder and order pictures varied trial by trial according to a random trial sequence. Based on the most recent ERP studies (Nessler et al., 2005; Woollams et al., 2008; Kurilla and Gonsalves, 2012) involving cognitive disfluency or fluency, one hypothesis was that the early divergences of triggered ERP components between the disorder and order conditions would begin at approximately 100–800 ms at the parietal electrodes, and order pictures (fluent) would elicit more positive (less negative) ERPs than disorder pictures. However, the opposite hypothesis should not be precluded, i.e., that disorder pictures might elicit more positive ERPs than order pictures based on evidence reported by Leynes and Addante (2016).

In summary, in the present study, from a cognitive perspective, by adopting the ERP method, we aimed to examine the elicited real-time neural signals of disorder and order perception when participants process physical environmental and basic visual disorder and order pictures in an irrelevant red or green rectangle detection task, and we further attempted to test the hypothesis of cognitive disfluency in disorder perception.

## Materials and Methods

### Participants

Twenty-nine healthy college students were recruited from University of Jinan in China, and were paid 50¥ for their participation. All participants reported normal or corrected normal vision. The experiment was approved by the Institutional Review Board of the School of Education and Psychology at the University of Jinan, and each participant signed the informed written consent. In the statistical analysis, data from five participants were discarded because the EEG segments comprised less than 30%. Data of the remaining twenty-four participants (12 female, age range 17–19 years old, *M* = 18.79 years, *SD* = 0.78) were used for analysis.

### Apparatus and Stimuli

Participants were asked to sit approximately 65 cm away from a computer screen (a 19-inch monitor, 1024 × 768 pixels, 85 Hz) and keep their heads on a headrest and their eyes focused on the center of the screen during the test session, except during rest periods between blocks. The experimental stimuli were presented using E-prime software, version 2.0, against a red or green background.

#### Basic Visual Order/Disorder Pictures

The visual order/disorder stimuli were the pictures used in the study by Kotabe et al. (2016b) (Figure 1, left). The size of these pictures was 600 × 398 pixels. A separate group of 23 participants (11 female, age range 17–19 years old, *M* = 20.73 years, *SD* = 2.66) was first shown all 60 pictures one by one, and they were asked to evaluate the order/disorder level of each picture on a 7-point semantic differential scale anchored by the options of very disorderly to very orderly. A paired *t* test revealed that the rating score for the disorder pictures (*M* = 2.64, *SD* = 1.54) was significantly lower than that for the order pictures (*M* = 5.12, *SD* = 1.53) (*t*(22) = -10.53, *p* < 0.001).

#### Physical Environmental Order/Disorder Pictures

A total of 60 pictures collected from daily life were used in Hu Peng’s study as the experimental stimuli (Figure 1, right). These pictures were then scaled to a fixed size of 600 × 398 pixels. Additionally, 23 participants were asked to evaluate the order/disorder level of each picture on a 7-point semantic differential scale anchored by the options of very disorderly to very orderly. A paired *t* test revealed that the rating score for the disorder pictures (*M* = 2.12, *SD* = 1.13) was significantly lower than that for the order pictures (*M* = 5.74, *SD* = 1.28) (*t*(22) = -22.21, *p* < 0.001).

### Experimental Design and Procedure

The experimental design was 2 (order type: disorder vs. order) × 3 (hemisphere: left vs. central vs. right).

Each trial began with the presentation of a central fixation cross (angle 0.8°) for a varied period of 500–750 ms, followed then by an order or disorder picture with a green or red rectangle around the picture outside that lasted for 3000 ms. The participants were asked to notice the contents of the picture and press the “F” key for the red rectangle or the “J” key for the green rectangle, counterbalanced between the participants.

After 12 practice trials, the participants completed two blocks of 224 experimental trials. The pictures in the first block were basic visual disorder/order pictures, and the second block contained physical environmental disorder/order pictures. Each picture was presented twice, and all of the pictures in each block were randomly presented.

### ERP Recordings and Data Analysis

Continuous EEG was recorded by used 64 electrodes mounted in an elastic cap (Electro-Cap International, Inc.) and connected to the left mastoid. The data were removed offline and then re-referenced to the average of the left and right mastoids (M1 and M2). Vertical (VEOG) and horizontal (HEOG) electro-oculograms were recorded with bipolar channels from sites above and below the midpoint of the left eye and next to the outer can thus of each eye. Mild skin abrasions were created to reduce the electrode impedance to less than 5 kΩ. The EEG was bandpass filtered from 0.05 to 100 Hz, amplified with a gain of 500, and stored on a computer disk at a sample rate of 1000 Hz (Syn-Amps 4.5, Neuroscan, Inc.).

The continuous EEG signal was corrected for blink artifacts using an eye-movement reduction algorithm and was segmented into one epoch: from 300 ms prior to the presentation of the stimulus to 3000 ms after the presentation of the stimulus. The epochs were digitally filtered (low pass = 30 Hz, high pass = 1 Hz) and were baseline corrected against the mean voltage during the 300-ms prestimulus period. The trials were automatically eliminated if the voltage in the epoch exceeded ± 125 μV.

We measured six components elicited by the basic visual disorder/order stimuli, which were identified over the posterior electrodes (P7/P8, PO7/PO8, Pz, and POz): N1 (60–100 ms), P1 (100–150 ms), N2 (150–200 ms), P2 (200–250 ms), N3 (250–350 ms) and P3 (350–600 ms). We also measured four components that were identified over the frontal electrodes (F7/F8, FT7/FT8, Fz and FCz): N1 (100–180 ms), P1 (180–240 ms), N2 (240–350 ms) and P3 (350–500 ms). Six components were measured that were elicited by the physical environment disorder/order stimuli; these components were identified over the posterior electrodes (P7/P8, PO7/PO8, Pz and POz): N1 (60–80 ms), P1 (80–120 ms), N2 (120–180 ms), P2 (180–250 ms), N3 (250–350 ms) and P3 (350–600 ms). Five components were also measured that were identified over the frontal electrodes (F7/F8, FT7/FT8, Fz and FCz): N1 (80–150 ms), P1 (150–200 ms), N2 (200–300 ms), P3 (300–450 ms) and N4 (450–600 ms). For each ERP component, a separate statistical analysis was conducted; for details, see Section “Results”.

## Results

In the irrelevant rectangle identification analysis, each participant’s response accuracy was approached to 90%, and the average correct accuracy of twenty-four participants was 98.55% (SD = 11.95). In the EEG analysis, the error trials based on the behavioral responses were excluded (less than 30%).

### The ERP Components Elicited by the Basic Visual Order/Disorder Pictures

**FIGURE 2:**
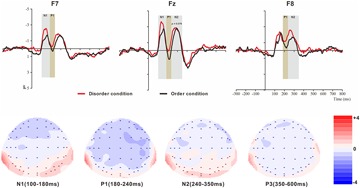
Top: Average ERP waveforms at frontal F7, Fz, and F8 electrodes elicited by centrally presented basic visual disorder (red line) or order (black line) pictures. The significant differences between the disorder and order conditions are indicated by the gray (negative differences) and earthy yellow (positive differences) rectangles, respectively. Time = 0 ms indicates the stimulus onset. Bottom: Topographic maps of the mean voltage amplitudes for the difference waveforms (disorder condition minus order condition) matched to the time window of each component elicited by the stimuli (notably the frontal locations). Increased negativity is shown in blue, while increased positivity is shown in red.

**FIGURE 3:**
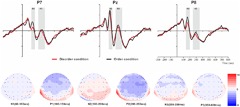
Top: Average ERP waveforms at posterior P7, Pz, and P8 electrodes elicited by centrally presented basic visual disorder (red line) or order (black line) pictures. The significant differences between the disorder and order conditions are indicated by the gray (negative differences) rectangles, respectively. Time = 0 ms indicates the stimulus onset. Bottom: Topographic maps of the mean voltage amplitudes for the difference waveforms (disorder condition minus order condition) matched to the time window of each component elicited by the stimuli (notably the posterior locations). Increased negativity is shown in blue, and increased positivity is shown in red.

For the ERP components elicited by the basic visual order/disorder pictures (frontal electrode voltage average from F7/F8, FT7/FT8, Fz and FCz (Figure 2) and the posterior electrode voltage averaged from P7/P8, PO7/PO8, Pz and POz) (Figure 3), two-way repeated ANOVA with order type (disorder vs. order) and hemisphere (left vs. central vs. right) was conducted separately.

#### The ERP Components at the Frontal Electrodes

##### N1

The analysis of the N1 (latency range of 100–180 ms) mean amplitude revealed a highly significant main effect of order type (*F*(1, 23) = 4.64, *p* < 0.05, $\eta_{p}^{2}$ = 0.17), with a significantly higher absolute mean voltage in the disorder condition (-2.03 μV) than in the order condition (-1.58 μV). The main effect of hemisphere also approached significance (*F*(2, 46) = 8.46, *p* < 0.001, $\eta_{p}^{2}$ = 0.27), with a significantly higher absolute mean voltage in the electrodes in the central area (-2.33 μV) than in the left (-1.48 μV) and right (-1.61 μV) hemispheres (*p*s < 0.01). No interaction effect was found (*F*(2, 46) = 1.30, *p* > 0.05). Nonetheless, the planned contrasts of order type revealed that there were significant differences at the electrodes in the central area (*M*_disorder_ = -2.61 μV vs. *M*_order_ = -2.05 μV, *F*(1,23) = 4.77, *p* < 0.05, $\eta_{p}^{2}$ = 0.17) and the electrodes in the left hemisphere (*M*_disorder_ = -1.73 μV vs. *M*_order_ = -1.24 μV, *F*(1,23) = 5.25, *p* < 0.05, $\eta_{p}^{2}$ = 0.19).

##### P1

The analysis of P1 (latency range of 180–240 ms) mean amplitude revealed a highly significant main effect of order type (*F*(1, 23) = 8.32, *p* < 0.01, $\eta_{p}^{2}$ = 0.27), with significantly higher absolute mean voltage in the disorder condition (-0.85 μV) than in the order condition (-0.21 μV). The main effect of hemisphere also approached significance (*F*(2, 46) = 7.50, *p* < 0.01, $\eta_{p}^{2}$ = 0.25), with a significantly higher absolute mean voltage in the electrodes in the right region (-1.12 μV) than in the central region (-0.02 μV) (*p* < 0.01). No interaction effect was found (*F*(2, 46) = 2.43, *p* > 0.05). Nonetheless, the planned contrasts of order type revealed that there were significant differences at the electrodes in the central area (*M*_disorder_ = --0.45 μV vs. *M*_order_ = -0.042 μV, *F*(1,23) = 8.43, *p* < 0.01, $\eta_{p}^{2}$ = 0.27) and the electrodes in the left (*M*_disorder_ = -1.73 μV vs. *M*_order_ = -1.24 μV, *F*(1,23) = 3.24, *p* = 0.085, $\eta_{p}^{2}$ = 0.12, marginally significant) and right hemispheres (*M*_disorder_ = -1.45 μV vs. *M*_order_ = -0.79 μV, *F*(1,23) = 6.92, *p* < 0.05, $\eta_{p}^{2}$ = 0.23).

##### N2

The analysis of N2 (latency range of 240–350 ms) mean amplitude revealed a highly significant main effect of order type (*F*(1, 23) = 7.61, *p* < 0.05, $\eta_{p}^{2}$ = 0.25), with significantly higher absolute mean voltage in the disorder condition (-1.82 μV) than in the order condition (-1.44 μV). The main effect of hemisphere also approached significance (*F*(2, 46) = 6.55, *p* < 0.01, $\eta_{p}^{2}$ = 0.22), with a significantly higher absolute mean voltage at the electrodes in the central area (-2.15 μV) than in the left (-1.36 μV) and right (-1.38 μV) hemispheres (*p*s < 0.01). No interaction effect was found (*F*(2, 46) = 0.99, *p* > 0.05). Nonetheless, the planned contrasts of order type revealed that there were significant differences only at the electrodes in the central area (*M*_disorder_ = -2.34 μV vs. *M*_order_ = -1.95 μV, *F*(1, 23) = 3.39, *p* = 0.079, $\eta_{p}^{2}$ = 0.13) and the electrodes in the right hemisphere (*M*_disorder_ = -1.68 μV vs. *M*_order_ = -1.08 μV, *F*(1,23) = 8.68, *p* < 0.01, $\eta_{p}^{2}$ = 0.27).

##### P3

The analysis of P3 (latency range of 350–600 ms) mean amplitude revealed no significant main effect of order type. The main effect of hemisphere approached significance (*F*(2, 46) = 3.37, *p* < 0.05, $\eta_{p}^{2}$ = 0.13), with a significantly higher absolute mean voltage at the electrodes in the central area (1.19 μV) than in the right (-0.34 μV) hemisphere (*p* < 0.05). No interaction effect was found (*F*(2, 46) = 0.80, *p* > 0.05). The planned contrasts of order type revealed no significant differences at the left, central or right electrodes.

#### The ERP Components at the Posterior Electrodes

##### N1

The analysis of N1 (latency range of 60–100 ms) mean amplitude revealed neither order type and hemisphere main effects (*F*(1, 23) = 0.70, *p* > 0.05; *F*(2, 46) = 0.22, *p* > 0.05) nor an interaction effect (*F*(*F*(2, 46) = 1.36, *p* > 0.05). The planned contrasts of order type revealed no significant differences at the left, central or right electrodes.

##### P1

The analysis of P1 (latency range of 100–150 ms) mean amplitude revealed no order type or hemisphere main effects (*F*(1, 23) = 1.62, *p* > 0.05; *F*(2, 46) = 0.44, *p* > 0.05). The interaction effect was marginally significant (*F*(2, 46) = 3.20, *p* = 0.05, $\eta_{p}^{2}$ = 0.12). Further simple comparisons showed that, at the electrodes in the right hemisphere, the absolute voltage in the order condition (-1.05 μV) was significantly higher than the voltage in the disorder condition (-0.63 μV) (*p* < 0.05).

##### N2

The analysis of N2 (latency range of 150–200 ms) mean amplitude revealed a highly significant main effect of order type (*F*(1, 23) = 10.27, *p* < 0.01, $\eta_{p}^{2}$ = 0.31), with a significantly lower absolute mean voltage in the disorder condition (-0.35 μV) than in the order condition (-0.96 μV). The main effect of hemisphere also approached significance (*F*(2, 46) = 3.93, *p* < 0.05, $\eta_{p}^{2}$ = 0.15), with a significantly higher absolute mean voltage at the electrodes in the central region (-1.01 μV) than in the left region (-0.04 μV) (*p* < 0.05). No interaction effect was found (*F*(2, 46) = 0.25, *p* > 0.05). Nonetheless, the planned contrasts of order revealed that there were significant differences only at the electrodes in the central area (*M*_disorder_ = -0.68 μV vs. *M*_order_ = -1.34 μV, *F*(1,23) = 6.88, *p* < 0.05, $\eta_{p}^{2}$ = 0.23) and the electrodes in the left (*M*_disorder_ = 0.23 μV vs. *M*_order_ = -0.30 μV, *F*(1,23) = 10.78, *p* < 0.01, $\eta_{p}^{2}$ = 0.32) and right hemispheres (*M*_disorder_ = -0.59 μV vs. *M*_order_ = -1.23 μV, *F*(1,23) = 7.38, *p* < 0.05, $\eta_{p}^{2}$ = 0.24).

##### P2

The analysis of P2 (latency range of 200–250 ms) mean amplitude revealed no significant main effect of order type (*F*(1, 23) = 0.89, *p* > 0.05). The main effect of hemisphere approached significance (*F*(2, 46) = 4.17, *p* < 0.05, $\eta_{p}^{2}$ = 0.15), with a significantly higher absolute mean voltage at the electrodes in the central (2.98 μV) than in the left (1.92 μV) and right (1.82 μV) hemispheres (*p*s < 0.05). No interaction effect was found (*F*(2, 46) = 0.87, *p* > 0.05). The planned contrasts of order type revealed no significant differences at the left, central or right electrodes.

##### N3

The analysis of N3 (latency range of 250–350 ms) mean amplitude revealed a highly significant main effect of order type (*F*(1, 23) = 6.45, *p* < 0.05, $\eta_{p}^{2}$ = 0.22), with significantly higher absolute mean voltage in the disorder condition (1.28 μV) than in the order condition (0.92 μV). The analysis of mean amplitude revealed no significant hemisphere main effect of order type (*F*(2, 46) = 0.82, *p* > 0.05) or interaction effect (*F*(2, 46) = 0.38, *p* > 0.05). Nonetheless, the planned contrasts of order type revealed that there were significant differences only at the electrodes in the central area (*M*_disorder_ = 1.27 μV vs. *M*_order_ = 0.86 μV, *F*(1,23) = 5.11, *p* < 0.05, $\eta_{p}^{2}$ = 0.18) and the electrodes in the left (*M*_disorder_ = 1.09 μV vs. *M*_order_ = -0.79 μV, *F*(1,23) = 5.77, *p* < 0.05, $\eta_{p}^{2}$ = 0.20) and right hemispheres (*M*_disorder_ = 1.49 μV vs. *M*_order_ = 1.13 μV, *F*(1,23) = 4.56, *p* < 0.05, $\eta_{p}^{2}$ = 0.17).

##### P3

The analysis of P3 (latency range of 350–600 ms) mean amplitude revealed no significant main effect of order type (*F*(1, 23) = 1.00, *p* > 0.05). The main effect of hemisphere approached significance (*F*(2, 46) = 12.87, *p* < 0.001, $\eta_{p}^{2}$ = 0.36), with a significantly higher absolute mean voltage at the electrodes in the central area (1.80 μV) than in the left (1.44 μV) and right (1.07 μV) hemispheres (*p* s < 0.05). No interaction effect was found (*F*(2, 46) = 1.79, *p* > 0.05). The planned contrasts of order type revealed no significant differences at the left, central or right electrodes.

### The ERP Components Elicited by the Physical Environment Order/Disorder Pictures

**FIGURE 4:**
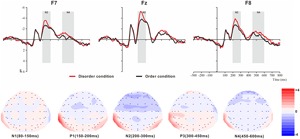
Top: Average ERP waveforms at frontal F7, Fz, and F8 electrodes elicited by centrally presented physical environment disorder (red line) or order (black line) pictures. The significant differences between the disorder and order conditions are indicated by the gray (negative differences) rectangles, respectively. Time = 0 ms indicates the stimulus onset. Bottom: Topographic maps of the mean voltage amplitudes for the difference waveforms (disorder condition minus order condition) matched to the time window of each component elicited by the stimuli (notably the frontal locations). Increased negativity is shown in blue, and increased positivity is shown in red.

**FIGURE 5:**
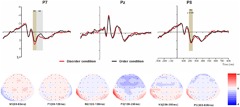
Top: Average ERP waveforms at posterior P7, Pz, and P8 electrodes elicited by centrally presented physical environment disorder (red line) or order (black line) pictures. The significant differences between the disorder and order conditions are indicated by the gray (negative differences) and earthy yellow (positive differences) rectangles, respectively. Time = 0 ms indicates the stimulus onset. Bottom: Topographic maps of the mean voltage amplitudes for the difference waveforms (disorder condition minus order condition) matched to the time window of each component elicited by the stimuli (notably the posterior locations). Increased negativity is shown in blue, and increased positivity is shown in red.

For the ERP components elicited by the physical environment order/disorder pictures (frontal electrode voltage averaged from F7/F8, FT7/FT8, Fz and FCz (Figure 4) and posterior electrode voltage averaged from P7/P8, PO7/PO8, Pz and POz) (Figure 5), two-way repeated ANOVA with order type (disorder vs. order) and hemisphere (left vs. central vs. right) was conducted separately.

#### The ERP Components at the Frontal Electrodes

##### N1

The analysis of N1 (latency range of 80–150 ms) mean amplitude revealed no significant main effect of order type (*F*(1, 23) = 0.008, *p* > 0.05). The main effect of hemisphere approached significance (*F*(2, 46) = 5.17, *p* < 0.01, $\eta_{p}^{2}$ = 0.18), with significantly higher absolute mean voltage at the electrodes in the central area (-1.61 μV) than in the left (-0.99 μV) and right (-1.05 μV) hemispheres (*p*s < 0.05). No interaction effect was found (*F*(2, 46) = 0.001, *p* > 0.05). The planned contrasts of order type revealed no significant differences at the left, central or right electrodes.

##### P1

The analysis of P1 (latency range of 150–200 ms) mean amplitude revealed neither order type and hemisphere main effects (*F*(1, 23) = 2.17, *p* > 0.05; *F*(2, 46) = 1.78, *p* > 0.05) nor an interaction effect (*F*(2, 46) = 0.92, *p* > 0.05). The planned contrasts of order type found no significant differences at the left, central or right electrodes.

##### N2

The analysis of N2 (latency range of 200–300 ms) mean amplitude revealed a highly significant main effect of order type (*F*(1, 23) = 10.10, *p* < 0.01, $\eta_{p}^{2}$ = 0.31), with significantly higher absolute mean voltage in the disorder condition (-3.33 μV) than in the order condition (-2.65 μV). The main effect of the hemisphere also approached significance (*F*(2, 46) = 8.75, *p* < 0.01, $\eta_{p}^{2}$ = 0.28), with a significantly higher absolute mean voltage at the electrodes in the central area (-3.88 μV) than in the left (-2.45 μV) and right (-2.64 μV) hemispheres (*p*s < 0.01). No interaction effect was found (*F*(2, 46) = 1.72, *p* > 0.05). Nonetheless, the planned contrasts of order revealed that there were significant differences only at the electrodes in the central area (*M*_disorder_ = -4.32 μV vs. *M*_order_ = -3.45 μV, *F*(1,23) = 9.34, *p* < 0.01, $\eta_{p}^{2}$ = 0.29) and the electrodes in the left (*M*_disorder_ = -2.71 μV vs. *M*_order_ = -2.19 μV, *F*(1,23) = 6.00, *p* < 0.05, $\eta_{p}^{2}$ = 0.21) and right hemispheres (*M*_disorder_ = -2.97 μV vs. *M*_order_ = -2.30 μV, *F*(1,23) = 9.13, *p* < 0.01, $\eta_{p}^{2}$ = 0.28).

##### P3

The analysis of P3 (latency range of 300–450 ms) mean amplitude revealed neither order type and hemisphere main effects (*F*(1, 23) = 2.59, *p* > 0.05; *F*(2, 46) = 0.82, *p* > 0.05) nor an interaction effect (*F*(2, 46) = 0.32, *p* > 0.05). The planned contrasts of order type revealed no significant differences at the left, central or right electrodes.

##### N4

The analysis of N2 (latency range of 450–600 ms) mean amplitude revealed a marginally significant main effect of order type (*F*(1, 23) = 3.45, *p* = 0.076, $\eta_{p}^{2}$ = 0.13), with significantly higher absolute mean voltage in the disorder condition (-0.98 μV) than in the order condition (-0.62 μV). The main effect of hemisphere also approached marginal significance (*F*(2, 46) = 3.06, *p* = 0.056, $\eta_{p}^{2}$ = 0.12), with higher absolute mean voltage at the electrodes in the central region (-1.13) than in the left (-0.44 μV) and right (-0.83 μV) hemispheres. No interaction effect was found (*F*(2, 46) = 0.036, *p* > 0.05). Nonetheless, the planned contrasts of order type revealed that there were marginally significant differences only at the electrodes in the left (*M*_disorder_ = -0.63 μV vs. *M*_order_ = -0.24 μV, *F*(1,23) = 3.01, *p* = 0.096, $\eta_{p}^{2}$ = 0.12) and right hemispheres (*M*_disorder_ = -1.01 μV vs. *M*_order_ = -0.65 μV, *F*(1,23) = 3.01, *p* = 0.096, $\eta_{p}^{2}$ = 0.12).

#### The ERP Components at the Posterior Electrodes

##### N1

The analysis of N1 (latency range of 60–80 ms) mean amplitude revealed no order type main effect (*F*(1, 23) = 0.10, *p* > 0.05). The main effect of hemisphere approached significance (*F*(2, 46) = 4.69, *p* < 0.05, $\eta_{p}^{2}$ = 0.17), with a significantly lower absolute mean voltage at the electrodes in the central (-1.43 μV) than in the right region (-2.08 μV) (*p* < 0.05). The interaction effect was marginally significant (*F*(2, 46) = 3.17, *p* = 0.051, $\eta_{p}^{2}$ = 0.12). Further simple comparisons revealed no significant differences between the disorder and order conditions at the left, central or right electrodes.

##### P1

The analysis of P1 (latency range of 80–120 ms) mean amplitude revealed neither order type and hemisphere main effects (*F*(1, 23) = 0.38, *p* > 0.05; *F*(2, 46) = 2.02, *p* > 0.05) nor an interaction effect (*F*(2, 46) = 0.99, *p* > 0.05). The planned contrasts of order type revealed no significant differences at the left, central or right electrodes.

##### N2

The analysis of N2 (latency range of 120–180 ms) mean amplitude revealed no order type main effect (*F*(1, 23) = 1.05, *p* > 0.05). The main effect of hemisphere approached significance (*F*(2, 46) = 9.75, *p* < 0.001, $\eta_{p}^{2}$ = 0.30), with a significantly higher absolute mean voltage at the electrodes in the central region (-1.94 μV) than in the left (-0.26 μV) and right regions (-0.54 μV) (*p*s < 0.05). The interaction effect was significant (*F*(2, 46) = 4.67, *p* < 0.05, $\eta_{p}^{2}$ = 0.17). Further simple comparisons revealed no significant differences between the disorder and order conditions at the left, central or right electrodes.

##### P2

The analysis of P2 (latency range of 180–250 ms) mean amplitude revealed no significant main effects of order type and hemisphere (*F*(1, 23) = 0.78, *p* > 0.05; *F*(2, 46) = 1.81, *p* > 0.05). The interaction effect was significant (*F*(2, 46) = 8.72, *p* < 0.01, $\eta_{p}^{2}$ = 0.28). Further simple comparisons revealed significant differences between the disorder and order conditions at the electrodes in the left (*M*_disorder_ = 2.67 μV vs. *M*_order_ = 2.24 μV, *p* < 0.05) and right (*M*_disorder_ = 2.46 μV vs. *M*_order_ = 2.09 μV, *p* = 0.082, marginally) hemispheres.

##### N3

The analysis of N3 (latency range of 250–350 ms) mean amplitude revealed a highly significant main effect of order type (*F*(1, 23) = 3.62, *p* < 0.05, $\eta_{p}^{2}$ = 0.18), with a significantly higher absolute mean voltage in the disorder condition (1.01 μV) than in the order condition (0.69 μV). The main effect of hemisphere approached significance (*F*(2, 46) = 9.65, *p* < 0.001, $\eta_{p}^{2}$ = 0.30), with a significantly lower absolute mean voltage at the electrodes in the central area (-0.13 μV) than in the left (1.22 μV) and right (1.45 μV) hemispheres (*p*s < 0.05). No interaction effect was found (*F*(2, 46) = 1.01, *p* > 0.05). Nonetheless, the planned contrasts of order type revealed a significant difference only at the electrodes in the left hemisphere (*M*_disorder_ = 1.43 μV vs. *M*_order_ = 1.01 μV, *F*(1, 23) = 12.18, *p* < 0.05, $\eta_{p}^{2}$ = 0.35).

##### P3

The analysis of P3 (latency range of 350–600 ms) mean amplitude revealed neither order type and hemisphere main effects (*F*(1, 23) = 0.04, *p* > 0.05; *F*(2, 46) = 0.84, *p* > 0.05) nor an interaction effect (*F*(2, 46) = 0.84, *p* > 0.05). The planned contrasts of order type revealed no significant differences at the left, central or right electrodes.

## Discussion

In the current study, we measured ERP responses elicited by disorder and order stimuli (basic visual and physical environmental types), while the participants performed an irrelevant red or green rectangle detection task. In general, we observed that at each measured time interval, the ERPs elicited by order stimuli were more positive (less negative) in amplitude than those evoked by the disorder stimuli at most of the frontal electrodes (represented by F7/F8, FT7/FT8, Fz and FCz), whereas at the most of the posterior electrodes (represented by P7/P8, PO7/PO8, Pz and POz), the opposite pattern was observed. This study makes two theoretical contributions. First, these data revealed the neural underpinnings of disorder and order perception, extending our understanding of the nature of disorder and order. Second, this study contributes to the cognitive fluency literature. Cognitive fluency has been manipulated by varying the clarity of stimuli (Whittlesea et al., 1990), the foreground/background color combinations (e.g., Reber and Schwarz, 1999), font (e.g., Westerman et al., 2003), and the pre-experimental experience (e.g., comparing famous and non-famous faces; Nessler et al., 2005). The current research indirectly expands the research on disorder and order stimuli in cognitive fluency.

At the frontal electrodes, for both the basic visual or physical environmental stimuli, the order ERP amplitudes were more positive (less negative) than the disorder ERP amplitudes, consistent with most prior evidence indicating that more fluent stimuli are associated with more positive ERPs (Nessler et al., 2005; Woollams et al., 2008; Kurilla and Gonsalves, 2012). Notably, in general, the latency of ERP components triggered by the physical environmental pictures was slightly earlier than the latency of those triggered by basic visual stimuli (see Figure 2–5). It is possible that the physical environmental stimuli were presented after the basic visual stimuli, and mere exposure to basic visual stimuli promoted fluent processing of the physical visual stimuli to some extent.

At the frontal electrodes, for the basic visual stimuli, significant differences in response to images depicting order versus disorder were found from 100 to 180 ms (N1), from 180 to 240 ms (P1) and from 240 to 350 ms (N2). The early sensory-evoked N1 and P1 components (anterior selection positivity) are traditional ERP markers of early selection (Hillyard et al., 1998). The first difference (N1) was strongest at the left-midfrontal electrodes. Evoked potentials during this time interval have been shown to reflect attention orienting or the early attention process (Griffin et al., 2001). This early sensory effect tended to be larger in the more difficult condition, particularly over the frontal scalp (Lange and Schnuerch, 2014). As mentioned in several works by Kotabe (2014); Kotabe et al. (2016a,b), processing of disordered stimuli is more disfluent and difficult than processing of ordered stimuli. Thus, in the present results, the disorder pictures elicited larger negative amplitudes than the order pictures, revealing that, in contrast to the order pictures, the disorder pictures attract our curiosity and attention easily because of their disfluency and the difficulty in processing them. In the current study, the disorder pictures elicited smaller P1 amplitudes than the order pictures at the left, middle and right frontal locations, and the time interval was brief. We suggest that this finding might reflect the participants’ sustained attention to the disorder pictures. The third ERP difference between the disorder and order conditions was that N2 peaked during the 240- to 350-ms interval. Prior research has proposed that N2 is a negative ERP typically evoked 180 to 325 ms following the presentation of a specific visual or auditory stimulus, and it appears to be closely associated with the cognitive processes of perception and selective attention (Patel and Azzam, 2005). Several distinct N2 potentials have been characterized (Näätänen and Picton, 1986); N2a is an anterior cortical distribution evoked by either conscious attention to or ignorance of a deviating stimulus (Pritchard et al., 1991); N2b is a sharp negative component with a front-central or central electrode often preceding P3, observed only during conscious stimulus attention; N2c arises frontally and centrally during classification tasks (Patel and Azzam, 2005). According to previous studies and the N2 component illustrated in Figure 2, the disorder pictures triggered a larger N2b deflection than the order pictures. Early findings suggested that N2b is clearly associated with controlled processes because its occurrence is dependent on focal attention to the stimulus (Näätänen and Picton, 1986), and N2b is elicited by unexpected stimuli (Duncan-Johnson and Donchin, 1982). As we mentioned at the beginning of this paper, order is comforting because it is associated with predictability (Harmonjones and Harmonjones, 2002). Disorder, in contrast, is aversive because it prevents people from anticipating what will happen next (Inzlicht and Kang, 2010; Hirsh et al., 2012); thus, it is reasonable to speculate that, in contrast to the order pictures, the disorder pictures, as unexpected stimuli, elicited a larger N2b component.

At the frontal electrodes, for the physical environmental stimuli, significant differences in response to disorder images versus order images were found from 200 to 300 ms (N2b) and from 450 to 600 ms (N4). The larger N2b amplitude of disorder than order pictures might also be produced by the unpredictability or unexpected nature of the disorder stimuli. One finding that was initially surprising was the N4 divergence at the left-right electrodes. It is now well established that N4 (N400) is a negative EEG potential evoked by a meaningful stimulus and is usually modulated by the semantic relatedness of consecutive linguistic and non-linguistic stimuli, such as images (Kutas and Federmeier, 2000; Kanske and Kotz, 2007). We suggest that since the physical environmental disorder and order pictures contain semantic information, and the disorder pictures convey more information than the ordered stimuli, the disorder pictures resulted in larger N4 amplitudes. No early sensory difference was found for the N1 (80–150 ms) and P1 (150–200 ms) components, possibly because the objects in the disorder and order conditions were highly similar (see Figure 1); the only difference was that the objects were positioned differently, so the attention allocation was equal between the two conditions.

At the posterior area, first, the amplitude divergences between the disorder and order conditions revealed by the early N1 (time interval of basic visual stimuli: 60–100 ms; time interval of physical environmental stimuli: 60–80 ms) and P1 (time interval of basic visual stimuli: 100–150 ms; time interval of physical environmental stimuli: 80–120 ms) did not approach significance, proving our assumption and previous research findings. Previous neuropsychological studies, including a study by Lorteije et al. (2006), have indicated that the timing of the N1 deviation in the posterior visual areas coincides with spatial frequency and orientation (Arakawa et al., 2000), size, and eccentricity (Busch et al., 2004). The triggered N1 and P1 components indicate that, for both the physical environment stimuli that we created and the basic visual stimuli created by Kotabe et al. (2016a), low-level visual features were quality matched between the disorder and order pictures, affording reliable experimental materials for future studies.

However, unexpectedly, for both the basic visual stimuli and the physical environment stimuli, the order pictures elicited more negative amplitudes than the disorder pictures, except for the deferred latency of ERP components in the posterior area. For the basic visual stimuli, the differences occurred at the later N2 (150–200 ms) and N3 (250–350 ms) components in the left, middle and right areas. For the physical environmental stimuli, the differences occurred at the P2 (180–250 ms) and N3 (250–350 ms) components in the left and right regions. Although the elicited ERP pattern was consistent with Voss and Paller (2010) and Leynes and Addante (2016) (randomly presented stimuli), the finding that more fluent stimuli elicited a more negative ERP contradicted the elicited ERP pattern at the frontal electrodes. This issue will need to be explored in future ERP studies specifically designed to address it. As with the stimuli presentation manipulation of Leynes and Addante (2016), to acquire a more thorough understanding of disorder/order perception, future studies should consider the multiple contributions of the manner in which the stimuli are presented (blocked verse randomly).

In the current study, we mainly interpreted the results from the cognitive fluency perspective. Other interpretation might also be existed. For example, Chae and Zhu (2014) proposed that orderliness could affect an individual’s sense of personal control, and they claimed that, compared with an organized environment, a disorganized environment increases self-regulatory failure. However, in the current study, the irrelevant red rectangle identification task was not suitable for revealing the ERP response to the processing of self-control or self-regulatory failure of disorder perception. Thus, it remains an open question whether disorder actually causes a low sense of personal control and consequently increases self-regulatory failure. We believe that ERPs are a powerful tool that could provide insight into these issues in future studies.

## Author Contributions

KL and FL designed the experiment and wrote the manuscript. XQ and HY performed the experiments and analyzed the data. GC and MZ revised the manuscript.

## Conflict of Interest Statement

The authors declare that the research was conducted in the absence of any commercial or financial relationships that could be construed as a potential conflict of interest.
